# Application of neural mass models to major depressive disorder

**DOI:** 10.1186/1471-2202-14-S1-P26

**Published:** 2013-07-08

**Authors:** Natalia Z Bielczyk

**Affiliations:** 1 Donders Centre for Neuroscience, Radboud University, Nijmegen, 6525AJ, The Netherlands

## 

Major depression disorder (MDD) is a serious medical condition of a global lifetime prevalence exceeding 16% in the US [1], and growing share in the global burden of disease, anticipated to reach the first place in the WHO rating by 2030 [2]. Nevertheless, neural underpinnings of MDD are far from being explained.

Systems generating affective states yield a variety of decisions, actions and behaviors in a response to the same sensory stimuli for different individuals. In particular, when such a system falls into an aberrant activity, it can lead to impairment in mood regulation and result in a mood disorder, such as MDD.

**Figure 1:**
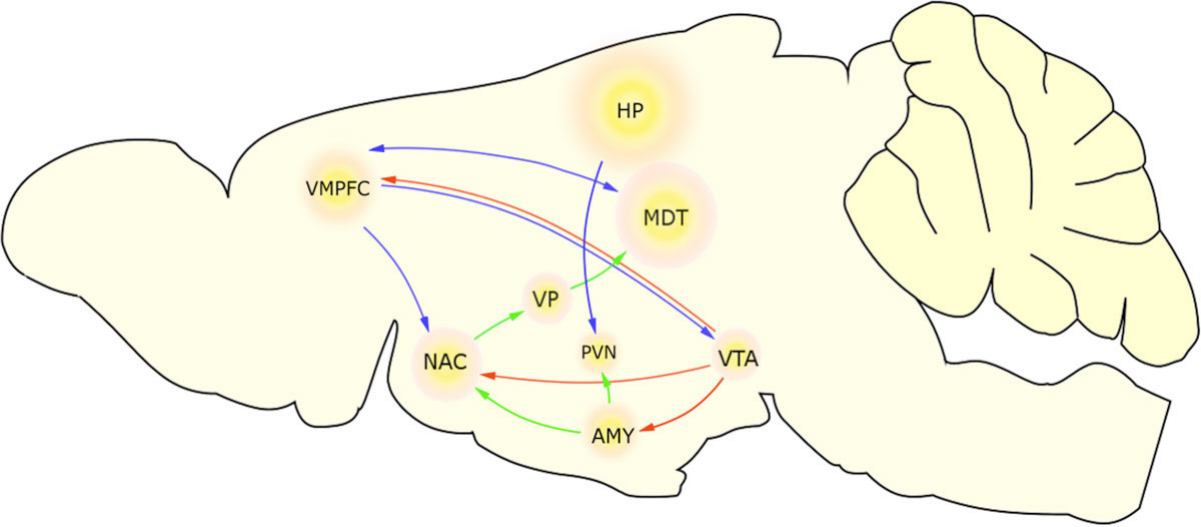
**Circuit involved in mood in a rat**.

The disorder develops from processes ongoing on a system level rather than from a single region dysfunction[3]. Neuroanatomical abnormalities in hippocampus (HP) and amygdala (AMY) highly correlate with depression, both in humans and animals [3,4]. HPA axis, represented by paraventricular nucleus (PVN) in the brain, is also involved as the 'stress' circuit [5]. PVN gets afferent projections from both HP and AMY. Additionally, recent research in optogenetics indicates basal ganglia to be a great contributor to the mood as a part of corticomesolimbic loop (a.k.a. 'mood' or 'affective' loop). The loop goes from ventral medial prefrontal cortex (VMPFC) to medial dorsal thalamus (MDT) [6]. Ventral tegmental area (VTA) is another important node in the network, as strongly connected with NAC and AMY, and a source of dopaminergic signal. Figure 1 shows this whole circuit in a rat, with basal ganglia reduced to nucleus accumbens (NAC) as the major input nucleus, and ventral pallidum (VP) as the major output.

Although the phenomenon of falling into MDD is hard to catch, both experimentally and computationally, one can bring a new insight into the difference between healthy and depressed brain by building dynamical models based on the above circuit. For this purpose, neural mass models are promising for three reasons. First, imaging experiments indicate under- or over-activation in the nodes of the circuit in depression, whereas oscillations specific to MDD are not found yet, which suggests rate coding in this system. Second, imaging studies allow for model validation. Third, since triggering activity in one of the nodes in the network may cause the whole dynamical system drift to a new fixed point, and changes in multiple loci in the network can trigger the same global state, the model demonstrates that multiple scenarios ongoing in the underlying network may lead to the same behavioral phenotype. Wilson-Cowan model[7] is of special interest in this study. With this approach, one shows that multiple mechanisms can allow for MDD to develop in a given individual. This research draws attention to the computational aspect of affective disorders, and proposes possible explanation for the difficulty encountered by clinicians in the domain of coupling patients suffering from MDD with the appropriate treatments.
